# Terahertz Radiation Modulates Neuronal Morphology and Dynamics Properties

**DOI:** 10.3390/brainsci14030279

**Published:** 2024-03-14

**Authors:** Shaoqing Ma, Peng Ding, Zhengxuan Zhou, Huilong Jin, Xiaoli Li, Yingwei Li

**Affiliations:** 1 School of Information Science and Engineering, Yanshan University, Qinhuangdao 066004, China; msq@stumail.ysu.edu.cn (S.M.); dpsl@stumail.ysu.edu.cn (P.D.); zhouzx@stumail.ysu.edu.cn (Z.Z.); 2 College of Engineering, Hebei Normal University, Shijiazhuang 050024, China; 13131145063@163.com; 3 Hebei Key Laboratory of Information Transmission and Signal Processing, Qinhuangdao 066004, China; 4 State Key Laboratory of Cognitive Neuroscience and Learning, Beijing Normal University, Beijing 100875, China

**Keywords:** terahertz radiation, neurobiological effects, neurons, morphology, dynamics properties

## Abstract

Terahertz radiation falls within the spectrum of hydrogen bonding, molecular rotation, and vibration, as well as van der Waals forces, indicating that many biological macromolecules exhibit a strong absorption and resonance in this frequency band. Research has shown that the terahertz radiation of specific frequencies and energies can mediate changes in cellular morphology and function by exciting nonlinear resonance effects in proteins. However, current studies have mainly focused on the cellular level and lack systematic studies on multiple levels. Moreover, the mechanism and law of interaction between terahertz radiation and neurons are still unclear. Therefore, this paper analyzes the mechanisms by which terahertz radiation modulates the nervous system, and it analyzes and discusses the methods by which terahertz radiation modulates neurons. In addition, this paper reviews the laws of terahertz radiation’s influence on neuronal morphology and kinetic properties and discusses them in detail in terms of terahertz radiation frequency, energy, and time. In the future, the safety of the terahertz radiation system should be considered first to construct the safety criterion of terahertz modulation, and the spatial resolution of the terahertz radiation system should be improved. In addition, the systematic improvement of the laws and mechanisms of terahertz modulation of the nervous system on multiple levels is the key to applying terahertz waves to neuroscience. This paper can provide a platform for researchers to understand the mechanism of the terahertz–nervous system interaction, its current status, and future research directions.

## 1. Introduction

**Figure 1:**
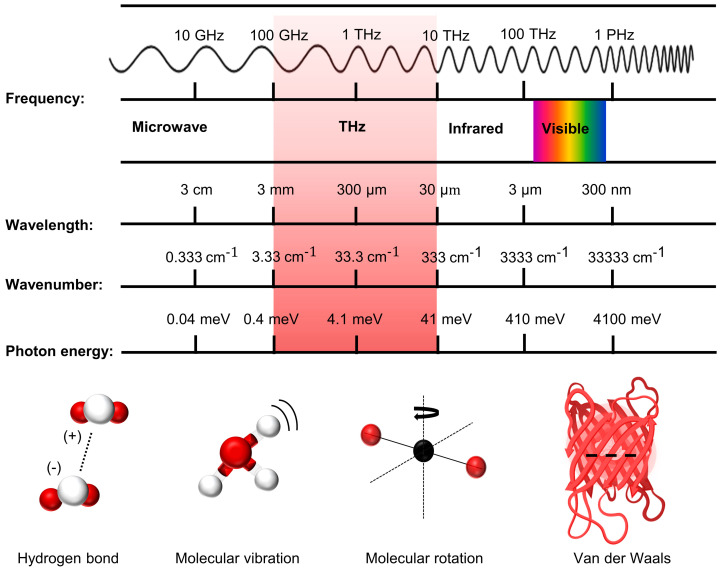
Terahertz bands in the electromagnetic spectrum and the associated intermolecular forces.

The frequency of terahertz radiation lies between microwave and infrared light and is an essential region in the transition from macroscopic electronics to microscopic photonics, known as the “terahertz gap” [1,2,3]. Terahertz waves, officially named in the late 1980s [4], are usually electromagnetic waves with a frequency of 0.1–10 THz, a wavelength of 30–3000 μm, a wave number of 3.3–333 cm^−1^, a period of 0.1–10 ps, a photon energy of 0.4–41 mev, and a temperature in the range of 4.8–478 K [5,6,7], as shown in Figure 1. Terahertz waves have photonic and electronic properties, their photon energy is low, they do not ionize matter, and they have high penetration and fingerprint spectral properties [8,9]. Therefore, terahertz waves are widely used in security detection, superconducting materials, food, and medicine [10,11,12,13]. In addition, because many energy level jumps for macromolecular interactions are in the terahertz band, terahertz waves have a wide range of applications in biomedical imaging [14,15].

Furthermore, many interactions between significant biomolecules occur within the terahertz frequency range, where absorption and resonance are pronounced [16]. Additionally, terahertz radiation falls within the scope of hydrogen bonding, molecular rotation, vibration, and van der Waals forces, signifying that even basic molecules can effectively absorb terahertz radiation [17]. Moreover, various substantial biomolecules (such as proteins, DNA, RNA, lipids, and even carbohydrates) possess distinct vibrational patterns and complex intramolecular interactions, like protein folding and DNA double-stranding [18,19]. These findings indicate the prevalence of low-frequency terahertz vibrations in living organisms [20], and life’s functionality is intricately associated with low-frequency terahertz vibrations. Changes in terahertz dynamics dictate functional states, encompassing processes like ion transport and ligand binding [21,22].

A number of studies have indicated that manipulating low-frequency vibrations within living organisms can influence the functional states of biological systems [23,24]. As a result, terahertz waves with specific frequencies and energies can directly interact with proteins, inducing coherent excitations and producing non-thermal effects [7,15]. Although the energy of terahertz radiation is insufficient to break chemical bonds between molecules, both theoretical and experimental evidence suggests that terahertz radiation can engage with hydrogen bonds in proteins, provoking low-frequency molecular vibrations. This, in turn, leads to alterations in protein conformation and functional characteristics [25,26,27]. Therefore, terahertz radiation can facilitate changes in cellular structure and function by triggering non-linear resonance effects in proteins and DNA.

The mechanism by which terahertz interacts with the nervous system is currently unclear, but there are some studies suggesting that terahertz bioeffects are produced by affecting cell membranes [28,29]. The cell membrane is an important biological barrier for biological cells to maintain integrity and internal homeostasis, and it is also the first cell structure that terahertz radiation acts on [30,31]. When terahertz radiation of different frequencies passes through the cell membrane, the molecules on the membrane will produce different time delays and vibrational absorption, showing the dielectric response properties of the cell membrane in the terahertz band [32]. The transmembrane transport of molecules and ions across the cell membrane maintains the basic metabolism of the cell, the homeostatic environment, and the function of a variety of biological activities, and it is also the only way for material exchange to occur between the internal and external environments of the cell [33,34]. Terahertz radiation can cause changes in the distribution of bioelectric fields in the cellular region, which, in turn, affects the state of the voltage-gated ion channels on the cell membrane to open and close. Bo et al. found through modeling simulation that 2.5 THz radiation can open pressure-controlled calcium ion channels and promote calcium ion transmembrane endocytosis [35]. Among them, terahertz radiation can prompt calcium ions to cross the energy potential barrier within the channel, which is the physical process that causes calcium ion transmembrane transport. In addition, terahertz radiation can also affect the opening of cell membrane active transport ion channels and transmembrane transport of macromolecules [36,37,38]. Following this mechanism, terahertz radiation with particular frequencies and energies can govern the morphology and function of neurons at the molecular level.

While some studies have shown that terahertz radiation can affect the nervous system of animals, the regulatory mechanism remains unclear. Additionally, current research primarily focuses on cellular levels, which is an overly simplistic approach that cannot comprehensively reveal the patterns of terahertz radiation’s impact on the nervous system. There is a lack of systematic research from multiple perspectives. Furthermore, it is necessary to consider the safety of terahertz radiation in regulating the nervous system. Guidelines for the safe terahertz regulation of the nervous system should be established to indicate the safe parameter range for the terahertz modulation of the nervous system. Furthermore, the research involves a diverse range of subjects, including chicken embryo neurons, hippocampal neurons, and African clawed toad embryo neurons, among others, and the terahertz radiation systems used in experiments vary significantly. This diversity makes it challenging to compare experimental results. In order for terahertz radiation to be used as a non-invasive neuromodulation technique, a comprehensive review of research related to terahertz neurobiological effects is needed, and current challenges and future research directions need to be identified.

This article provides a comprehensive review of the mechanisms underlying terahertz radiation’s regulation of neurons and its impact on neuronal morphology and function. It extensively discusses the methods employed for the terahertz radiation modulation of neurons, the influence of terahertz radiation on neuronal development, and the effects of terahertz radiation on neuronal function and animal behavior. Finally, it explores the current challenges and prospects from three perspectives: the safety of terahertz radiation, the compatibility of terahertz devices, and the regulatory mechanisms of the nervous system.

## 2. The Mechanisms of Terahertz Radiation Modulation on Neurons 

While the energy of individual terahertz photons is as low as a milli-electronvolt and lacks direct ionizing damage similar to X-rays [14], increasing the intensity of terahertz radiation leads to a series of biological effects on neurons. From a physics perspective, the biological effects of terahertz radiation on neurons fundamentally originate from the thermal and non-thermal effects of terahertz radiation [7]. The thermal effect results from the strong absorption of terahertz radiation by a large number of water molecules in neurons or the neuronal environment. In contrast, the non-thermal effect primarily arises from the nonlinear resonance effects induced by terahertz radiation on biological macromolecules in neurons.

### 2.1. Thermal Effect

The generation of terahertz radiation’s thermal effects is primarily due to the absorption of terahertz radiation by neurons and its conversion into thermal energy. The neuronal absorption of terahertz radiation is mainly related to water molecules and biological macromolecules. Since the water content in neurons is much higher than that of biological macromolecules, it serves as the major chromophore in the terahertz frequency range, resulting in the dominant contribution of water to the thermal effect [9]. Water possesses several unique properties, including the ability to form hydrogen bonds with adjacent water molecules, creating a dynamic hydrogen bond network [39]. The stretching and bending vibrational modes between the molecules within this network occur within the terahertz frequency range (with the most prominent resonant frequencies at 5.6 and 1.5 THz) [40,41,42]. When neurons are exposed to terahertz waves, the vibrational modes of the hydrogen bonds are excited, causing resonance. This disrupts the dynamic equilibrium of the water molecule’s network structure, resulting in the strong absorption of terahertz radiation by water. The absorbed terahertz radiation energy is transformed into the kinetic energy of the random motion of water molecules, increasing the frequency of collisions between water molecules, thus generating thermal energy [7]. In the absence of photochemical processes and phase changes, the continuous accumulation of heat will directly lead to an increase in neuronal temperature [9].

The thermal effects of terahertz radiation on neurons can lead to changes in neuronal morphology and function. These changes have a dual nature and are primarily associated with the extent of neuronal temperature increase and the duration of elevated temperatures. The prolonged exposure of neurons to high-power terahertz radiation, leading to a significant and sustained increase in temperature, can result in disrupted neuronal growth, dehydration effects, neuronal morphological damage, neuronal stress responses, and, in severe cases, structural protein denaturation and neuronal cell death [9,43,44]. However, when the duration of elevated temperature within neurons is on the millisecond scale, it can affect neuronal calcium homeostasis, induce the generation of action potentials in neurons, impact neuronal synaptic transmission, and affect firing rates [45], and these effects are reversible.

### 2.2. Non-Thermal Effect

Some studies have indicated that certain changes in gene expression and individual behavior observed in biological systems exposed to terahertz radiation cannot be explained by thermal effects (without significant temperature elevation or changes in heat shock protein expression) [46,47]. For these effects observed in terahertz radiation biological systems, they are generally attributed to non-thermal effects. The theory of the non-thermal effects of terahertz radiation was initially proposed by Frohlich and others in 1971, and more recent research results suggest that many biological macromolecules have energy levels associated with rotation, oscillation, and torsion in the terahertz frequency range. Specific frequencies and energy levels of terahertz waves can be directly coupled with proteins, inducing coherent excitations and resulting in non-thermal effects [48,49].

Although the energy of terahertz radiation is insufficient to break chemical bonds between molecules, both theoretical and experimental evidence suggests that terahertz radiation can interact with hydrogen bonds in proteins, inducing low-frequency molecular vibrations, consequently altering the conformation and functional characteristics of proteins. Additionally, radiation can induce non-thermal structural changes in protein crystals [25,26,27]. Research by Aleksandrov and others, using computer simulations, revealed that terahertz radiation can disrupt the natural dynamics of a local strand separation in double-stranded DNA, thereby affecting DNA function [50]. Furthermore, studies have pointed out that terahertz radiation can precisely control proton transfer processes in base pair hydrogen bonds, thereby regulating DNA demethylation [51,52]. These studies indicate that terahertz radiation can mediate changes in cellular morphology and function by exciting nonlinear resonance effects in proteins and DNA. Based on this mechanism, terahertz radiation of specific frequencies and energy levels can impact the morphology and function of neurons.

## 3. Methods for Terahertz Radiation Regulation of Neurons 

### 3.1. Radiation System for Terahertz Regulation of Neurons

The research on the terahertz radiation regulation of neuron morphology and function is also influenced by the radiation system. When investigating the regulatory patterns of the terahertz radiation on the nervous system, it is essential to understand how the nervous system responds to different terahertz radiation parameters. Therefore, it is necessary to adjust the output frequency and power of the terahertz radiation system to generate various radiation protocols. Additionally, the terahertz radiation system needs to be compatible with the biological experimental platform. However, current technology cannot always meet these requirements. Consequently, in research, different types of terahertz radiation systems are usually employed to achieve the adjustable power and frequency demands. For example, researchers frequently use both broadband and narrowband terahertz radiation systems. Broadband terahertz radiation systems cover a broader frequency range, making it easier to induce resonance in biological macromolecules. In contrast, when the controlling resonance peaks of biological macromolecules are identified, narrowband terahertz radiation systems are often chosen, which operate near the resonance peak.

Broadband terahertz systems generally use pulsed terahertz sources, which emit pulsed signals with durations on the order of picoseconds in the time domain and exhibit broad spectra in the frequency domain [49]. Pulsed broad-spectrum terahertz sources generated through optical methods typically employ femtosecond lasers as the pump light source [49]. These lasers excite different materials to produce terahertz waves using methods like photoconductive antennas, optical rectification, and gas plasma [53]. Among these techniques, the terahertz waves generated through photoconductive antennas primarily cover the lower frequency range and have relatively lower power levels. Optical rectification is currently an important method for producing high-energy single-pulse terahertz radiation. It uses femtosecond laser-pumped DSTMS organic crystals to generate terahertz radiation with single-pulse energies of up to 0.9 mJ [49,54]. Additionally, the spectrum of terahertz radiation produced through optical rectification is much broader than that of photoconductive antennas, spanning from 0.1 to 100 THz, covering the entire terahertz range [49]. Gas plasma terahertz sources are also common for generating pulsed broad-spectrum terahertz radiation. Compared to the methods mentioned above for generating terahertz waves through photoconductive and optical rectification, gas plasma terahertz sources can achieve ultra-broad terahertz spectra ranging from 0.1 to 30 THz [49,55]. However, since the medium used to generate terahertz radiation in gas plasma sources is unstable, the intensity of the produced terahertz radiation can also exhibit certain fluctuations [49], which may affect research on terahertz neurobiological effects.

When studying the effects of terahertz radiation on neuron morphology and function, the choice of terahertz radiation frequencies typically falls within the range of 0.1–5 THz because many energy levels of various biomolecules (proteins, DNA, RNA) are within this frequency range [18]. Furthermore, higher terahertz radiation frequencies can result in neuronal heating and induce thermal effects [43]. Therefore, the terahertz radiation power levels used in research are usually kept below 10 mW. Additionally, considering the compatibility of terahertz radiation systems with biological experimental platforms, it is desirable for these systems to have as compact a form factor as possible. Currently, the systems used for researching the impact of terahertz radiation on neuron morphology and function predominantly involve broadband terahertz radiation systems generated using photoconductive antennas. For instance, in the study investigating the effects of broadband terahertz radiation in minute quantities on neuron growth and development, the terahertz radiation system used had a frequency range of 0.3–3 THz, a maximum radiation power of up to 100 μW, and a repetition rate of 100 MHz. To ensure that an adequate amount of terahertz radiation reached the samples, the experimental process also included real-time measurements of terahertz radiation intensity through the culture dishes [56]. Additionally, M.V. Tsurkan and colleagues employed a broadband terahertz radiation system with a frequency range of 0.05–2 THz and a repetition rate of 50 MHz to study the impact of radiation on chicken embryonic neurons. They obtained terahertz waves with power levels of 11.1 and 1.07 mW through different terahertz filters [57]. Furthermore, M.I. Sulatsky and others used a broadband terahertz radiation system (0.1–2 THz) to investigate the impact of radiation power on chicken embryonic spinal cord ganglia [58].

When determining the resonance peaks of biological macromolecules in the controlled neurons, narrowband terahertz radiation systems are typically chosen around these resonance peaks. These narrowband terahertz radiation systems generally allow for the continuous emission of terahertz radiation and are produced using various methods, including free-electron lasers, gas lasers, quantum cascade lasers, backward wave oscillators, and avalanche diodes [49]. Terahertz sources based on free-electron lasers exhibit an output power that surpasses that of the photoconductive antenna method by more than six orders of magnitude, but they tend to have a larger footprint and higher production costs [49,59]. Terahertz gas lasers can also generate terahertz waves with a relatively high average power. By altering the type of gas and gas pressure, these lasers can produce terahertz radiation at different frequencies (approximately 0.9–7 THz). However, their conversion efficiency is low, and they tend to be bulky [60]. Quantum cascade lasers, based on semiconductor technology, can achieve output frequencies as low as 1.19 THz. They offer the advantage of being compact and suitable for integration but are not operable at room temperature [61]. Backward wave oscillators can adjust their output frequency by varying the acceleration voltage. Typically, their operating frequency is below 1.5 THz, and they provide an average output power in the milliwatt range [49,62]. Terahertz sources based on avalanche diodes are widely used due to their relatively high linear output power and compact design. They generally operate at frequencies below 1 THz and can achieve output powers of up to 100 mW [63].

When studying the effects of terahertz radiation on brain tissue slices or in vivo neuronal discharge characteristics, it becomes crucial to enhance the output power of terahertz sources. This is due to the significant absorption of terahertz radiation by cerebrospinal fluid, skin, and cranial bones. Additionally, to ensure compatibility with biological experimental platforms, terahertz radiation systems should be as compact as possible and operate at room temperature. Currently, the most widely used terahertz radiation source at the cellular, tissue, and in vivo levels is the avalanche diode-based terahertz source. For example, Zhang et al. utilized a terahertz radiation system operating at 0.1 THz with a power density of 2.65 mW/cm^2^ to confirm that terahertz radiation can modulate neuronal discharge characteristics by affecting ion concentrations inside neurons [64]. Furthermore, this study found that terahertz radiation at 0.138 THz and 2 mW promotes synaptic transmission in the hippocampal CA1 region. At the in vivo level, Miao et al. increased terahertz radiation power to 90 mW and observed that 0.14 THz radiation reduces anxiety and depression symptoms in mice while enhancing their social behavior [65]. Some studies have also used backward wave oscillators and quantum cascade lasers as terahertz sources, but these investigations are often limited to the cellular level. In such studies, due to the size and operating temperature of terahertz radiation sources, they are usually placed at specific locations in the experiments, and the terahertz radiation is transmitted to the samples using lenses and mirrors [66,67].

The response of neurons to terahertz radiation can be divided into real-time and delayed responses. Currently, most research focuses on analyzing the delayed response of neurons to terahertz radiation. However, studying the real-time response of neurons to terahertz radiation is also of great importance. In experiments, a real-time observation of how neurons’ morphology and functionality respond to terahertz radiation, as well as an accurate measurement of the terahertz radiation intensity reaching the sample, is crucial for ensuring the safety of constructing terahertz radiation protocols. Furthermore, due to variations in radiation samples, adjustments to the direction and beam area of terahertz radiation are often necessary. Given these requirements, there is currently a lack of terahertz radiation experimental platforms that are compatible with neuronal morphology recording and electrophysiology systems, as well as efficient methods for optimizing optical pathways for terahertz transmission direction and focusing.

### 3.2. Radiation Protocol for Terahertz Radiation Regulation of Neurons

**Figure 2:**
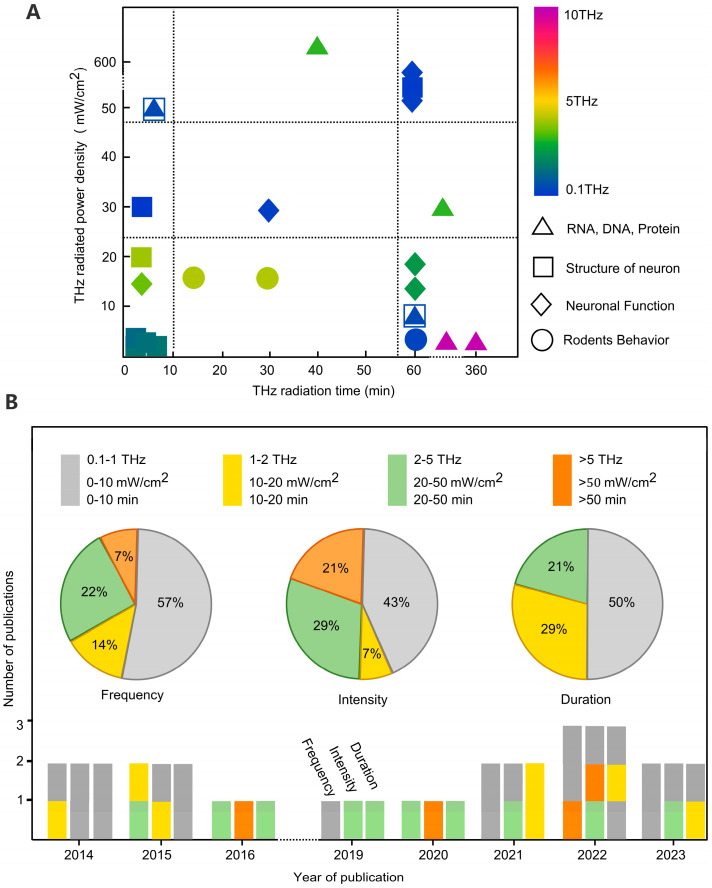
Analysis of the literature on terahertz neurobiological effects. (**A**) Heat map of the relationship between study objects and terahertz radiation protocols in the literature. (**B**) Analysis of terahertz radiation parameters in the literature related to terahertz neurobiological effects in the last decade.

Previous research has indicated that terahertz radiation can impact the morphology and functionality of neurons, and these effects are related to factors such as terahertz frequency, duration, and power [64,65,66]. Therefore, to further select appropriate terahertz radiation protocols, it is essential to analyze the distribution of research subjects and terahertz radiation protocols in the relevant literature. In retrieving the related literature, both Chinese and English keywords were initially set, including terahertz, neurons, nervous system, proteins, growth and development, action potentials, membrane potentials, receptors, etc. Subsequently, articles in both Chinese and English containing at least “terahertz” and one other keyword were searched in databases such as Web of Science, Google, PubMed, and China National Knowledge Infrastructure. Finally, parameters such as publication year, radiation frequency, power density, and exposure duration were summarized, and the results are shown in Figure 2.

Currently, most research aims to uncover terahertz neurobiological effects at the cellular level, with the majority of studies focusing on frequencies below 5 THz, particularly emphasizing the impact of low power densities on the nervous system. Over the past decade, 57% of the studies on the effects of terahertz radiation on neuronal morphology and functionality were conducted in the 0.1–1 THz range, and 93% in the 0.1–5 THz range. This concentration in the lower frequency range may be related to the vibrational rotation frequencies of biological macromolecules and is also influenced by the frequency of terahertz radiation sources (most commercial terahertz radiation sources are concentrated in the lower frequency range). Regarding terahertz radiation power, 43% of the studies had radiation power densities in the range of 0–10 mW/cm^2^, while 21% had power densities greater than 50 mW/cm^2^.

Through the analysis above, it is evident that research primarily focuses on two extremes: low- and high-power terahertz radiation. A higher radiation power often leads to neuronal heating, causing thermal effects [43]. On the other hand, a lower radiation power typically induces non-thermal effects on neurons, thereby influencing neuronal morphology and functionality [66,67]. Furthermore, some studies indicate that a lower power terahertz radiation can have positive impacts on neurons, such as promoting growth and enhancing synaptic transmission [64,67], while a higher power terahertz radiation can result in damage to neuronal morphology and function, leading to growth disturbances, dehydration, and cell death, among other effects [43,68]. In terms of terahertz radiation duration, studies generally involve single exposures lasting less than 50 min, with 50% of the research falling within the 0–10 min range. Most studies aim to mitigate the thermal effects of terahertz radiation on neurons by reducing exposure duration.

When studying the non-thermal effects of terahertz radiation on neurons, terahertz radiation influences neuronal morphology and functionality by exciting nonlinear resonances within the biomolecules present in neurons. Consequently, the frequency of terahertz radiation is crucial, as different biomolecules have distinct resonance peaks, allowing researchers to choose terahertz radiation frequencies based on the characteristics of the molecules being targeted. Some studies typically opt for broadband or narrowband terahertz radiation, with broadband terahertz radiation encompassing a wider frequency range, making it more likely to induce resonances in biomolecules [7]. However, once the resonance peaks for the specific biomolecules of interest are determined, researchers often select narrowband terahertz radiation close to these resonance peaks. Additionally, employing short-term accumulated terahertz radiation can further mitigate the thermal effects on neuronal responses.

The aforementioned analysis highlights that current research on terahertz neurobiological effects remains in an exploratory stage, characterized by a wide range of terahertz radiation power, duration, and frequency span. Selecting an appropriate terahertz radiation protocol poses a dilemma, as it needs to ensure sufficient power reaches neurons while minimizing temperature-related impacts. Choosing suitable terahertz radiation parameters can alleviate the negative effects of terahertz radiation on neurons. However, there is currently a lack of established criteria and methods for selecting the right terahertz radiation protocol.

## 4. The Impact of Terahertz Radiation on Neuronal Morphology and Dynamic Properties 

**Figure 3:**
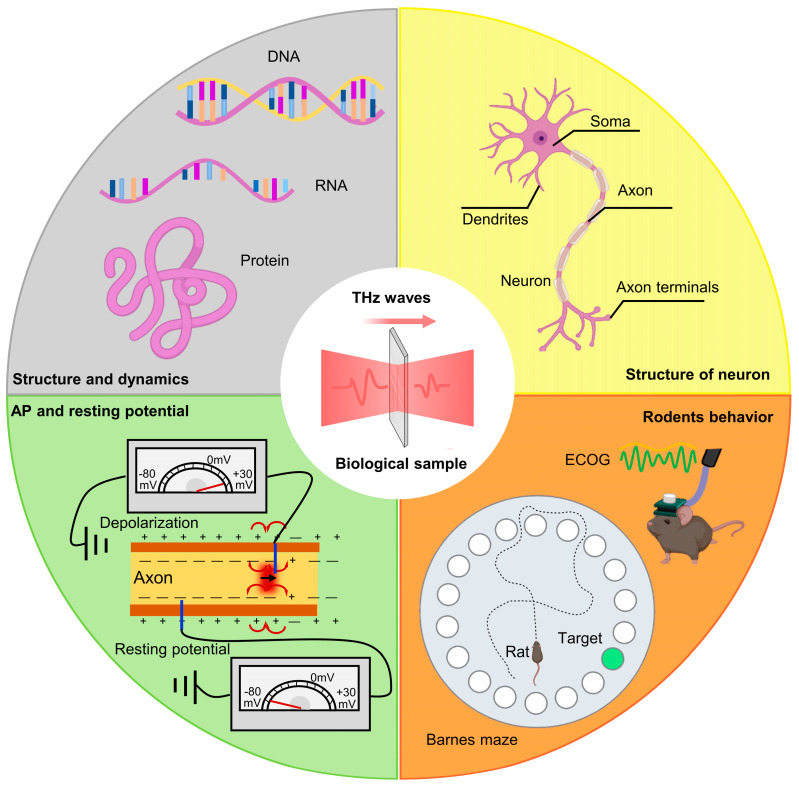
Research content related to terahertz neurobiological effects.

Recently, an increasing number of scholars have begun to focus on the neurobiological effects of terahertz radiation, as depicted in Figure 3. Some studies have shown that specific frequencies and energies of terahertz radiation can mediate changes in neuronal morphology and dynamic properties by exciting nonlinear resonances within proteins and DNA [25,26,27]. This section provides a detailed analysis of the impact of terahertz radiation on neuronal growth and development, the structure and composition of neuronal receptors, and neuronal action potential characteristics. And it discusses the correlation of these phenomena with the parameters of terahertz radiation (frequency, time, power). 

### 4.1. The Influence of Terahertz Radiation on the Growth and Development of Neurons

The cell body and processes of a neuron are fundamental structures that underpin the transmission and reception of information by neurons. At the tip of the neurite, there is an extension structure known as the growth cone [69,70], which serves as the driving organ of neuronal growth. The growth cone is capable of sensing signals in the environment and modifying the development process of the neuron, a process that also involves the polymerization and dissolution of cytoskeletal proteins [69,70]. Studies have shown that continuous terahertz radiation (0.094 THz, 1.86 W/cm^2^) for 30 min can disrupt the structure of actin [71], and that increasing the radiation power to 3.1 W/cm^2^ results in even more significant disintegration [71]. Subsequently, a reduction in both the power and duration of terahertz radiation was investigated, with the finding that after radiation at a power of 310 mW/cm^2^ for 3 min, the growth rate of neurons increased from 13.0 ± 0.9μm/min to 30.8 ± 3.9 μm/min, although the neuron temperature increased by more than 7 °C [72]. The relationship between temperature and microtubule assembly was analyzed, and it was determined that this phenomenon is associated with the increased temperature of the neuron [71,72]. Additionally, higher radiation powers (0.12 THz, 10 mW; 0.157 THz, 50 mW) have been shown to induce neuronal apoptosis, along with mitochondrial damage and increased lysosomes [73].

The above studies have shown that high-power terahertz radiation disrupts actin structural disassembly and causes neuronal death, mitochondrial damage, and increased lysosomes and that there is a significant increase in neuronal temperature [71,72,73]. It is evident that high-power terahertz radiation causes thermal effects on neurons and can negatively affect them [74,75]. Ma et al. constructed a thermal effect model for the interaction between terahertz and neurons and found that the temperature of neurons increases rapidly during terahertz radiation and stabilizes after a period of time. And, with the increase in the terahertz radiation power, the peak temperature in the neuron also increases [76]. Thus, terahertz radiation power and time are important factors that affect neurons.

To mitigate the effects of thermal radiation, some studies have chosen to reduce the power and duration of terahertz radiation exposure. For instance, Sulatsky et al. exposed chick embryo spinal cord neurons to terahertz radiation (0.1–2 THz) for 3 and 5 min. The results indicated that neuronal growth was inhibited at a power density of 928 mW/cm^2^; however, when the power density was reduced to 78 mW/cm^2^, terahertz radiation was found to promote neuronal growth. Furthermore, when the terahertz power density decreased to 19 mW/cm^2^, the growth of neurons in the irradiated group was 118.76 ± 11.3% greater than that in the control group [58]. To further reduce the thermal effects of terahertz radiation on neurons, a study employed terahertz radiation with a power density of 1.1 μW/cm^2^ to irradiate chick embryo sensory neurons for 10–12 days. The results showed that the growth of neurons in the irradiated group was 147 ± 22% greater than that in the control group [77]. These findings suggest that terahertz radiation power is a critical parameter affecting neuronal growth and development. Tsurkan et al. further confirmed the correlation between terahertz radiation power and neuronal growth and development. In their study, broad-spectrum pulsed terahertz radiation (0.05–2 THz) was used to irradiate chick embryo neurons for 3 min. They found no significant impact on neuronal growth and development when the terahertz radiation power density was 5 μW/cm^2^ and 50 μW/cm^2^ [57]. However, when the terahertz power density was reduced to 0.5 μW/cm^2^, terahertz radiation significantly promoted neuronal growth [57].

The frequency of terahertz radiation has also been found to influence the growth and development of neuronal processes. Some studies have observed the reflection spectrum of neurons exposed to terahertz radiation and found that the impact of terahertz radiation on the growth and development of neuronal processes is highly correlated with the absorption characteristics of neurons towards terahertz radiation [58]. The research conducted by Zhao and colleagues further supports this conclusion, as they demonstrated that exposure to 3.1 THz radiation can promote neuronal growth and synaptic formation by altering the dynamics of gene expression associated with neuronal development [67]. In summary, the frequency of terahertz radiation is also an important factor affecting neurons by the mechanism of absorption and resonance of terahertz radiation by neurons. Since both the neuron size and the terahertz wavelength are on the order of micrometers, they are equipped to interact [78,79,80]. In addition, the resonance peaks between biological macromolecules are different, so there are differences in the effects of different frequencies of terahertz radiation on neurons.

However, multiple studies have indicated that terahertz radiation can damage neurons. For instance, exposure to broadband terahertz radiation (0.09–0.16 THz) for 20 min has been shown to induce a dehydrating effect (reduction in cell volume) in snail neurons [68]. In these experiments, the dehydrating effect began as early as the first minute of terahertz radiation exposure and persisted for the duration of the 20 min irradiation period, and the effects continued for 10 min after the cessation of terahertz radiation [68]. Additionally, research has revealed that exposure to terahertz radiation (81.5 μm, 15 mW/cm^2^) at different stages of neuronal growth can lead to interruptions in the growth process and morphological damage, with the extent of damage being associated with the power and wavelength of the terahertz radiation [43]. Nevertheless, some studies have suggested that terahertz radiation does not affect neuronal growth and development [66]. In these studies, neurons were exposed to terahertz radiation of varying frequencies (0.16 THz, 0.17 THz), powers (10 mW, 50 mW), and irradiation times (10 min, 60 min), and no significant effects on neuronal process length and branch number were observed [66].

The above analysis reveals that the impact of terahertz radiation with different frequencies, energies, and irradiation times on neuronal growth and development varies (promotion, inhibition, no effect) and is highly related to the terahertz radiation protocol. The main radiation parameters and conclusions from related studies are presented in Table 1. However, to date, no research has pointed to the effects of short-term cumulative terahertz radiation on neuronal growth and development. Additionally, the growth and development of neurons is a dynamic and continuous process, and no studies have yet elucidated the influence of terahertz radiation on the dynamic growth and development of neurons, as well as the cumulative effects.

### 4.2. The Impact of Terahertz Radiation on Neuronal Membrane Permeability and Integrity

The neuronal membrane has a dual significance in neurons: firstly, as a barrier, it maintains the integrity of the cell and its internal compartments; secondly, as a medium for communication between the organelle and the external environment, it has a significant role in neuronal resting potential maintenance and electrical signal transduction [81,82]. Neuronal excitability depends not only on the difference in ion concentrations in-side and outside the cell but also on the neuronal membrane’s precise control and selective permeation [83,84].

Several studies have explored the effects of terahertz radiation at wavelengths of 130 μm and 150 μm on neuronal cell membrane integrity and permeability using a fluorescent dye that does not penetrate intact cell membranes (BCECF-AM) and a live cell assay [85]. It was found that some neurons were exposed to terahertz waves with a wavelength of 130 μm, and the permeability of the neuronal membrane was changed and successfully stained by the dye, with the proportion of stained cells proportional to the terahertz radiation power. The membrane potential of unstained neurons is about −60 mV, while the membrane potential of stained neurons is usually greater than −60 mV or close to 0 mV. But similar results do not appear for terahertz waves with a wavelength of 150 μm. Subsequently, researchers found that the terahertz effect at a wavelength of 130 μm caused reversible damage to the barrier properties of neuronal membranes due to reactive oxygen metabolites [85]. 

However, it has also been shown that terahertz waves have no significant effects on neuronal membranes [66]. In this study, seven different types of neurons were selected and exposed to different frequencies (0.16 THz, 0.17 THz), powers (10 mW, 50 mW), and irradiation times (10 min, 60 min) of terahertz radiation, and the results showed that terahertz radiation had no significant effects on the roughness of neuronal membranes.

From these studies, it is clear that terahertz radiation at a wavelength of 130 μm can reversibly alter the permeability of neuronal membranes and increase the resting potential of neurons. In addition, the permeability of neuronal membranes was correlated with the terahertz radiation power. However, the terahertz waves with a wavelength of 150 μm had no significant effect on the permeability of neuronal membranes. These phenomena suggest that the impact of terahertz waves on neuronal membrane permeability and integrity is not only related to the radiation power but also influenced by the wavelength. Terahertz waves can cause reversible changes in the properties of neuronal membrane barriers, acting as inducers to translocate bioactive compounds into cells. However, inappropriate terahertz radiation protocols may lead to neuronal dysfunction or death.

### 4.3. The Impact of Terahertz Radiation on Neuronal Dynamical Properties

Neurons possess the capacity to perceive stimuli and transmit excitation, with the propagation of neuronal excitation being achieved through the selective permeation of charged ions such as Ca^2+^, K^+^, and Na^+^ via ion channels [86]. Factors such as electric fields and magnetic fields often influence the opening and closing of ion channels, thereby affecting the neuronal membrane potential and action potentials. Guo and colleagues utilized Brownian Dynamics (BD) simulations to solve mathematical–physical models and investigated the spontaneous radiation produced by Ca^2+^ movement within calcium channels, as well as the impact of terahertz radiation on Ca^2+^ transport [87]. The study found that Ca^2+^ can emit terahertz radiation during its movement within calcium channels. Additionally, external terahertz radiation can accelerate Ca^2+^ transit through ion channels, and the acceleration effect is related to the frequency and amplitude of the terahertz radiation [87].

Sun and colleagues further experimentally demonstrated the correlation between terahertz radiation power and intraneuronal Ca^2+^ concentration. They found that terahertz radiation (0.094 THz) can regulate intraneuronal Ca^2+^ concentration by adjusting the secretion of ATP [88]. When the radiation power was 30 mW, the secretion of ATP increased by a factor of 5, and the peak Ca^2+^ concentration in neurons also increased; however, when the terahertz radiation power was increased to 60 mW, the secretion of ATP increased by a factor of 10, but the change in Ca^2+^ concentration in neurons was slow [88]. Furthermore, Titushkin and others also discovered that exposing neurons to terahertz radiation (0.094 THz, 18.6 kW/m^2^) led to a significant increase in the number of Ca^2+^ spikes in neurons [71].

Researchers from Tianjin University, Zhang et al., further explored the relationship between terahertz radiation, neuronal intracellular ion flow, and neuronal dynamical properties [64]. In their study, SD rat hippocampal neurons were irradiated with a terahertz light source with a frequency of 0.1 THz and a power density of 2.65 mW/cm^2^ for 5, 15, and 25 min. Subsequently, fluorescence detection techniques were used to observe changes in intraneuronal ion flow and membrane potential [64]. The results revealed that terahertz radiation for 15 and 25 min significantly induced depolarization in hippocampal neurons [64]. By detecting changes in the concentrations of Ca^2+^, K^+^, and Na^+^ in neurons, it was found that the concentrations of Ca^2+^ and Na+ increased, while K^+^ concentration decreased after radiation [64]. Additionally, terahertz radiation could enhance excitatory synaptic transmission and neuron firing rate [67]. At the neural network level, Ma et al. found that 0.138 THz waves improve hippocampal CA1 synaptic transmission efficiency, which is a continuous and slow process and highly correlated with terahertz radiation duration. After terahertz radiation, the effect of an increased synaptic transmission efficiency in the hippocampal CA1 area can last for more than 10 min [89].

The above analysis demonstrates that terahertz radiation can regulate neuronal discharge characteristics by affecting the concentration of intraneuronal ions. Table 2 lists studies on the impact of radiation on intraneuronal ion concentration and discharge characteristics. A wealth of experimental and modeling studies have shown that neuronal morphology can influence neuronal discharge characteristics; hence, changes in neuronal discharge dynamics are often accompanied by alterations in neuronal morphology. Investigating the impact of neuronal morphological parameters on neuronal discharge dynamics is crucial for understanding the relationship between neuronal structure and dynamical properties. To date, no studies have pointed out the correlation between neuron morphology and dynamical properties after terahertz radiation exposure. Moreover, there is a lack of research from the neuronal network perspective examining the regularities of terahertz radiation’s impact on neuronal synaptic transmission, synaptic function, and structural plasticity.

### 4.4. The Impact of Terahertz Radiation on Animal Behavior

The effect of terahertz radiation on rodent behavior reveals the macroscopic response of animals to terahertz waves. V. F. Kirichuk et al. placed the back skin of albino rats under continuous irradiation at 150.176–150.664 GHz THz for 30 and 60 min, and the total time spent by the rats passing through the maze and grooming their hair was significantly increased; signs of depression were found in the irradiated rats [91]. It was demonstrated that terahertz radiation affects the behavior and stress response of albino rats. Similarly, a study has pointed out that short-term exposure to terahertz waves (3.67 THz, 15 mW) negatively affects mice’s behavior. Thirty minutes of radiation had a substantial effect on the state of the experimental animals; the next day after radiation, mice showed a significant increase in anxiety levels [92]. The effects of terahertz radiation on rodent behavior are shown in Table 2.

## 5. The Current Challenges and Future Perspectives

### 5.1. Safety of Terahertz Regulation of the Nervous System

In recent years, research on terahertz neurobiological effects has begun, and the effects and regulatory mechanisms of terahertz waves on the nervous system are still unclear. However, the safety of terahertz radiation to the nervous system should be considered first, which requires establishing a set of safety evaluation criteria for terahertz radiation protocols and the selection criteria for radiation protocols. On the one hand, the effects of different terahertz radiation parameters on the nervous system can be explored through many biological experiments, and safe terahertz radiation parameters can be screened. This places high demands on the terahertz radiation system, while many biological experiments can also incur high costs. On the other hand, the response of the nervous system to different terahertz radiation parameters can be obtained by establishing a theoretical model of the interaction between terahertz waves and the nervous system and by studying the laws of the interaction between terahertz waves and the nervous system with different parameters. Also, some artificial intelligence algorithms (Artificial Neural Network, Deep Learning) can be used to establish the relationship between terahertz radiation parameters (frequency, power, radiation time) and the response of the nervous system (neuronal thermal damage rate, neuronal mortality). The range of safe terahertz radiation protocols is obtained through modeling. Finally, this range is further refined through some biological experiments.

### 5.2. Development of Terahertz Radiation System for Neurobiological Effects Research

In establishing the security of terahertz radiation protocols and the laws of terahertz wave modulation of the nervous system, it is necessary to obtain the nervous system’s response to different terahertz radiation parameters. This poses two problems. The first is the requirement of a terahertz radiation system with adjustable radiation power and frequency to generate different terahertz radiation protocols. However, few terahertz sources can meet such requirements; therefore, there is a need to develop terahertz sources with adjustable radiation power and frequency. Secondly, in cellular-level research, it is necessary both to allow neurons to survive in the right environment and that terahertz can radiate to the neurons in real time. And it is difficult to take care of all these at the same time in practical research. There is also a need to miniaturize the terahertz radiation system as much as possible, which will make the radiation system compatible with more biological experimental platforms. 

The future of terahertz waves as a neuromodulation technology, the directionality of the terahertz radiation system, and the radiation resolution are equally important. When intervening and treating neurological diseases, different diseases have different pathogenesis and involve different brain regions; for example, depression is associated with the prefrontal lobe and the hypothalamus [95], while Alzheimer’s disease is associated with brain regions such as the hippocampus and the cortex [96]. In order to treat different groups of people and diseases, it is necessary to adjust the direction of the radiation system according to the actual situation, so that it can radiate to the target brain area. The spatial resolution of the terahertz radiation system also needs to be improved if an area needs to be precisely regulated. The terahertz source and lens can be integrated into the robot arm, precisely regulating the angle and relative distance of the terahertz source and lens [97,98]. By precisely adjusting the angle and relative distance, terahertz waves’ radiation direction and resolution can be controlled.

### 5.3. The Mechanism of Terahertz Modulation of the Nervous System

In recent years, research on terahertz neurobiological effects has begun, and the effects and regulatory mechanisms of terahertz waves on the nervous system are still unclear. Current research is mainly focused on the cellular level, and there is a lack of systematic research at multiple levels. According to the mechanism of terahertz wave modulation of the nervous system, it can be found that the main target of its action is biological macromolecules. Therefore, the laws and mechanisms of terahertz regulation of the nervous system can be systematically studied at the gene and protein levels, at the cellular level, at the level of biological tissues, and at the level of living animals. In the study, genes responsive to terahertz radiation can first be screened out through gene sequencing and further verified at the cellular level through gene knockout and other techniques. Subsequently, some electrophysiological techniques can be used to carry out research at the in vivo level, and, finally, a series of behavioral experiments can be conducted to verify the modulation of the nervous system by terahertz radiation. Such a series of closed-loop research can comprehensively and rigorously reveal the mechanism of terahertz radiation regulation of the nervous system. These can be achieved by conducting a series of biological experiments. However, due to the lack of theoretical guidance and experience, it is not easy to find the exact mechanism of terahertz wave modulation. In order to solve this problem, a model of the nervous system dynamics can be constructed, and some algorithms (traceless Kalman filter algorithm) can be used to reverse estimate the parameter changes in the model. Finally, it can be validated by some biological experiments.

## 6. Conclusions

The biological effects of terahertz waves on specific types of neurons are still at the exploratory stage. Terahertz radiation of specific frequencies and energies mediates changes in neuronal morphology and function by exciting nonlinear resonances in proteins. Terahertz radiation can promote neuronal growth and development. Thus, terahertz radiation can be used as a treatment for diseases such as neurodevelopmental disorders or nerve damage. However, terahertz radiation can also cause neuronal growth disruption and death, which reminds people that the safety of terahertz radiation must be considered. The development of appropriate safety norms based on the damage thresholds of different terahertz radiation types gives a safe range of terahertz radiation parameters. On the other hand, terahertz radiation is also capable of modulating the kinetic properties of neurons. This is important because many neurological diseases are associated with abnormal neuronal discharges. Terahertz radiation can also be used as a treatment for neurodegenerative diseases. Since the target of terahertz radiation is a biomolecule, it has a very good specificity for the modulation of a particular biomolecule. In the future, terahertz radiation can be developed into a non-invasive neuromodulation technology with targets at the molecular level. The study of terahertz neurobiological effects also faces some challenges. The complexity of the terahertz systems used in the experiments is not conducive to a comparative analysis between experimental results because of the different subjects involved in the studies and the wide variation in the terahertz systems used in the experiments. Compact terahertz radiation sources for the study of neurobiological effects are in short supply so far, and the development of terahertz radiation systems with tunable parameters, orientation, and spatial resolution is fundamental for exploring the laws and mechanisms of terahertz modulation of the nervous system. In addition, a systematic study of the mechanisms and signaling pathways of terahertz interaction with the nervous system is needed at multiple levels. In the future, terahertz radiation can be developed into a non-invasive neuromodulation technique with targets at the molecular level.

## Figures and Tables

**Table 1:** The influence of terahertz radiation on the development and growth of neurons.

Cell In Vitro Study	Frequency	Intensity	Exposure Time	Effect	Reference
Hippocampal neurons Cortical neurons	0.16 THz; 0.17 THz	10 mW; 50 mW	6 min; 60 min	Will not adversely affect the development of neurons.	[66]
C57 mouse cortical neurons	not specified	70 μW/cm^2^	15 min; 3 h	Facilitate the formation of neuronal synapses.	[67]
Mouse embryonic cancer cells	0.094 THz	3.1 kW/cm^2^; 7.8 kW/cm^2^; 18.6 kW/cm^2^	60 min	The structure of neuronal actin protein is compromised.	[71]
Xenopus laevis embryonic neurons	0.094 THz	310 mW/cm^2^	3 min	Enhancing the growth rate of neurons.	[72]
Chicken spinal cord neurons at 10–12 days	0.05–1.2 THz	0.23–11.6 μW/m^2^	3 min	Facilitating or inhibiting the growth of neuronal processes.	[58]
Chicken embryonic spinal cord neurons	0.1–2 THz	1.1 μW/cm^2^	3–5 min	Promoting the growth and development of neurons.	[77]
Adult rats	0.09–0.16 THz	not specified	1–20 min	Neuronal dehydration atrophy.	[68]
Neurons	3.67 THz	15–20 mW/cm^2^	60 min	Impeding the growth of neurons.	[43]
Chicken embryo sensory neurons	0.05–2 THz	0.5–50 μW/m^2^	3 min	Neuronal growth is associated with radiation power.	[57]

**Table 2:** Effects of terahertz radiation on neuronal firing characteristics and rodent behavior.

Cell In Vitro Study	Frequency	Intensity	Exposure Time	Effect	Reference
C57 mouse cortical Neurons	not specified	70 μW/cm^2^	15 min; 3 h	Enhancing neuronal synaptic transmission and excitability.	[67]
Mouse embryonic cells	0.094 THz	3.1 kW/cm^2^; 7.8 kW/cm^2^; 18.6 kW/cm^2^	60 min	Increasing the number of Ca^2+^ peaks in neurons.	[71]
Neurons	3.67 THz	15–20 mW/cm^2^	60 min	Depolarization of neuronal membrane potential.	[43]
Mouse embryonic cells	0.094 THz	30 mW; 60 mW	30 min; 60 min	Modulating the concentration of Ca^2+^ within neurons.	[88]
SD rat hippocampal neurons	0.1 THz	2.65 mW/cm^2^	15 min; 25 min	Augmenting the concentrations of Ca^2+^ and Na^+^ within neurons.	[64]
Mouse pyramidal neurons	35–45 THz	10 mW; 30 mW	10–100 s	Augmenting the excitability of neurons.	[90]
Hippocampal CA1	0.138 THz	2 mW	60 min	Increased synaptic transmission efficiency.	[89]
Male albino rats	0.15 THz	0.2 mW/cm^2^	30 min; 60 min	Increased time for rats to pass through the maze, depression.	[91]
Male mice	3.6 THz	15 mW	15 min; 30 min	Mice showed a significant increase in anxiety levels.	[92]
Male albino rats	0.15 THz	3 mW/cm^2^	60 min	Induce signs of depression.	[93]
Male rats	0.167 THz; 0.144 THz	not specified	5 days	Maintain normal ability to explore new things; Increased anxiety, reduced appetite and sleep time.	[94]
Eight-week-old female C57BL/6 mice	0.14 THz	not specified	20 min	Enhanced anxiolytic, antidepressant, and social interaction.	[65]

## Data Availability

No new data were created or analyzed in this study. Data sharing is not applicable to this article.
